# Intravenous hMSCs Ameliorate Acute Pancreatitis in Mice via Secretion of Tumor Necrosis Factor-α Stimulated Gene/Protein 6

**DOI:** 10.1038/srep38438

**Published:** 2016-12-05

**Authors:** Zhigang He, Jie Hua, Daohai Qian, Jian Gong, Shengping Lin, Chenglei Xu, Ge Wei, Hongbo Meng, Tingsong Yang, Bo Zhou, Zhenshun Song

**Affiliations:** 1 Department of Hepatobiliary and Pancreatic Surgery, Shanghai Tenth People’s Hospital, School of Medicine, Tongji University, Shanghai, China

## Abstract

The administration of mesenchymal stem cells/multipotent mesenchymal stromal cells (MSCs) to enhance tissue repair is currently undergoing clinical trials. Some studies, including our previous work, have also revealed the beneficial effect of MSCs in severe acute pancreatitis (SAP); however, their mechanisms or mode of action remain controversial. In this study, we demonstrated that intravenously (i.v.)-administered human MSCs (hMSCs) remarkably promoted recovery from experimental SAP without significant engraftment of hMSCs in the damaged pancreas. Interestingly, we found that i.v.-administered hMSCs with knockdown of TSG-6 expression lost most of their anti-inflammatory effects and thus could not significantly ameliorate SAP. As expected, the effects of hMSCs were also duplicated by i.v. infusion of recombinant TSG-6. Furthermore, our results showed that the increase of oxidative stress, activation of the NLRP3 inflammasome and NF-*κ*B signaling in SAP was substantially inhibited following administration of hMSCs or TSG-6, which was dependent on the presence of CD-44 receptors in acinar cells. In conclusion, our study, for the first time, revealed that novel mechanisms are responsible for the immunomodulatory effect of i.v. hMSCs.

With an annual incidence of 13–45 cases per 100,000 persons worldwide and an unpredictable clinical course, acute pancreatitis (AP) is among the leading reasons for hospitalization from gastrointestinal diseases^1^. The severity of this disease ranges from a mild self-limited condition to a life-threatening situation with a high incidence of systemic complications. Among severe AP (SAP) patients with infectious pancreatic necrosis, systemic inflammatory response syndrome (SIRS) and multiple organ dysfunction syndrome (MODS), the overall mortality is reportedly as high as 30%^2^. Although the pathogenesis of SAP has not yet been elucidated^3^, it is generally believed that the onset and development of pancreatitis is primarily determined by intra-acinar events including premature activation of trypsinogen, release of inflammatory cytokines, damage-associated molecular patterns (DAMPs) and recruitment of inflammatory and immune cells. These events interact and reinforce each other, becoming a vicious circle and leading to further acinar cell injury and systemic inflammatory responses, which closely correlate with the severity and mortality of pancreatitis^4^,^5^,^6^. Recent advances in preclinical research have suggested that etiologic factors including oxidative stress^7^, NLR pyrin domain-containing protein-3 (NLRP3) inflammasome^8^ and nuclear factor kappa B (NF-κB)^9^,^10^ activation play particularly crucial roles in this disease, and blocking the vicious circle resulting from these factors could be the foundation of future therapeutic strategies.

For decades, different interventions have been proposed to treat, prevent, or at least limit, the development of SAP, but the mortality of patients with SAP has not declined significantly. Therefore, it is imperative that new effective approaches are developed. Numerous reports have described the beneficial effects in various disease models after administration of adult stem/progenitor cells, referred to as mesenchymal stem cells/multipotent mesenchymal stromal cells (MSCs)^11^,^12^,^13^,^14^. The valuable benefits of MSCs were initially attributed to their action of migration, engraftment and differentiation to repair tissues. However, remarkable anatomical structural repairs and functional improvements were increasingly observed even when only a small number or even no MSCs accumulated in the injured tissues, which suggests that the majority of the effects may be largely due to paracrine secretions or cell-to-cell contacts which have multiple effects involving modulation of inflammatory and immune responses^15^,^16^,^17^,^18^,^19^. Some studies^20^,^21^, including our previous work^22^,^23^, have revealed the potential therapeutic and preventive benefits of MSCs in SAP; however, the precise mechanisms through which MSCs exert their effect remains controversial. In addition, it is well known that most experimental studies which explore the effects of MSCs usually employ a rodent source. It has been suggested that the mechanisms of MSC-meditated immunosuppression may differ between rodent MSCs and human MSCs (hMSCs) in some experimental animal models but not in others, including the experimental allergic encephalomyelitis model of multiple sclerosis^24^. Before hMSCs are involved in clinical trials, it is critical to understand the mechanisms integral to the useful effects of hMSCs in SAP so that the human physiological response can be accurately predicted^25^.

Previous reports demonstrated that i.v.-infused hMSCs can significantly improve the outcome in experimental models of human disease and the therapeutic benefits of hMSCs are in part explained by cells being activated by signals from injured tissues and secreting the multifunctional anti-inflammatory protein tumor necrosis factor-α-stimulated gene/induced protein 6 (TSG-6/TNAIP6)^26^,^27^,^28^,^29^. SAP is always recognized as a typical pathological condition complicated by a systemic inflammatory response with a “cytokine storm”^30^, therefore we preferentially hypothesized that i.v.-infused hMSCs would exert their key effects without engraftment and differentiation in the injured pancreatic tissue and primarily via secretion of TSG-6, and elected to test this in a mouse model of SAP induced by sodium taurocholate (Na-taurocholate, NaT). Comparative analyses suggested that i.v.-administered hMSCs could modulate both the inflammatory process and tissue remodeling in this model via secreted TSG-6. Furthermore, we also explore the possible mechanisms for the action of TSG-6, implicating oxidative stress, NLRP3 inflammasome and NF- κB signaling depend on CD-44.

## Methods

### Ethics statement

The experimental protocol was approved by the institutional animal ethics committee of Tongji university, with animal care performed strictly according to the guide for the care and use of laboratory animals published by the US National Institutes of Health (NIH Publication No.85–23, revised 1996). All surgery was performed under pentobarbital anesthesia, and all efforts were made to minimize suffering.

### Induction of severe acute pancreatitis

Healthy wild-type male C57 BL/6 mice (18–24 g) were purchased from Shanghai SLAC Laboratory Animal Co., Ltd. (Shanghai, China). Animals were housed in temperature-controlled (22 °C) rooms with a 12-hour light/dark cycle, fed standard laboratory chow and given freely available water. The animals were acclimated for seven days before initiating the experiment. All surgical procedures were conducted by a single surgeon under aseptic conditions in the Laboratory Animal Unit. The animals were anesthetized by intraperitoneal injection of 2% pentobarbital (30 μg/g body weight). Skin preparation, and conventional immobilization, sterilization and covering with sterile drapes were all performed. With the aid of a dissecting microscope, a midline laparotomy was performed and the first loop of the duodenum was identified. The duodenum was flipped over to expose the pancreas and was immobilized with a 7–0 traction suture, and the papilla of Vater was identified. In addition, the proximal common bile duct was occluded at the liver hilum with a micro-clamp. A NaCl solution of 2% sodium taurocholate (Na-taurocholate, NaT; T6260; Sigma-Aldrich, St Louis, MO, USA) was retrogradely injected into the bile-pancreatic duct via the papilla of Vater over a 10-minute period using an infusion pump (Harvard Apparatus, Natick, MA, USA). Then the catheter, the micro-clamp, and the securing ligature were removed. The puncture site was closed using an 8–0 prolene suture, and the abdomen was closed in two layers with 6–0 prolene sutures. Subcutaneous buprenorphine hydrochloride injection was given immediately after wound closure^31^,^32^. Sham-operated mice underwent the same operation but without NaT injection. The animals were given free access to water and normal laboratory chow 6 hours after surgery (Fig. S1A,B).

### Cell preparation

Human bone marrow-derived MSCs were obtained commercially (Cyagen Biosciences, Sunnyvale, CA, USA). Passage four hMSCs were used in all experiments. According to the guidelines of the International Society for Cellular Therapy (ISCT), hMSCs can be characterized by expression of cell surface markers, analyzed by flow cytometry, and by their trilineage differentiation capacities. For flow cytometric analysis, hMSCs were labeled with the selected antibodies CD44-PE, CD-73-APC, CD90-FITC, CD105-PerCP-Cy5.5, CD45-PE, CD34-PE, CD11b-PE, CD19-PE, and HLA-DR-PE and corresponding isotype controls (562245; BD Biosciences Pharmingen, San Diego, CA, USA), and analyzed using a BD FACSCanto^™^ II flow cytometry system (BD Biosciences Pharmingen). Differentiation of hMSCs was evaluated using a human mesenchymal stem cell differentiation kit (HUXMA-90021; HUXMA-90031; HUXMA-90041; Cyagen Biosciences). Osteogenic and adipogenic differentiation assays were performed in six-well tissue culture plates, and chondrogenic differentiation assay was performed using a pellet culture system in 15 mL polypropylene culture tubes. All assays were conducted according to the manufacturer’s instructions. Pancreatic acinar cells (PACs) were obtained as described in a previous study^33^, and cultured with or without NaT (Sigma-Aldrich), and were also cocultured with or without murine macrophages (Raw264.7 cells). Human fibroblasts (hFs) were obtained from the American Type Culture Collection (ATCC, Manassas, VA, USA) and human umbilical vein endothelial cells (HUVECs) were purchased from Clonetics Cell Discovery Systems (San Diego, CA, USA); The human monocyte cell line THP-1 was obtained from the Institute of Biochemistry and Cell Biology (Shanghai, China).

### Transfection of hMSCs with TSG-6 siRNA

hMSCs were plated at 1.0 × 10^4^/cm^2^ in six-well plates and allowed to attach for 1 day, after which they were used as the target cells for transfection with 20 nM siRNA for TSG-6 (sc-39819; Santa Cruz Biotechnology, Santa Cruz, CA, USA) or scrambled siRNA (sc-37007; Santa Cruz Biotechnology) using a commercial kit (Lipofectamine RNAiMAX reagent; Invitrogen, Carlsbad, CA, USA). Six hours later, the medium containing the mixture for transfection was replaced with 2 mL fresh growth medium lacking antibiotics and the hMSCs were incubated for a further 16–20 hours. To confirm successful knockdown of TSG-6 expression, RNA was extracted from aliquots of the cells (RNeasy Mini kit; Qiagen, Valencia, CA, USA) and assayed for expression of TSG-6 by real-time RT-PCR.

### Experimental protocol

Considering that pancreatic injury/necrosis is relatively serious for mice being infused with NaT up to 6 h, and the similar severity of pancreatitis for patients in clinical also usually requires a standard treatment. Therefore, mice were treated with or without passage four hMSCs after 6 h NaT-induction and the beneficial effects for SAP were evaluated at 12, 24, 72 and 120 h (n = 4 or n = 6) after hMSCs treatment. And also SAP mice were randomized into a PBS-treated group (n = 8), MSC-treated group (n = 12), scr siRNA MSC-treated group (n = 8), TSG-6 siRNA MSC-treated group (n = 8) and recombinant human (rh) TSG-6-treated group (n = 8). The treatment groups then received either 2.0 × 10^6^ cells or 30 μg rhTSG-6 (R&D Systems Inc., Minneapolis, MN, USA) in a volume of 200 μL PBS which was slowly infused via the tail vein using a 27 G needle at 6 h after operation and a mouse treated with 200 μL PBS alone was used as the negative control. Before infusion, cells were resuspended with a pipette to ensure they were not aggregated, and mice were held in a tail vein injection restrainer with a warming water bath (45 °C), which restrained the animal and gently warmed the tail while allowing access to the tail vein. Sacrifice was performed after treatment, when blood samples were collected and centrifuged (400 × *g*, 30 min, 4 °C) to obtain serum, which was then stored at −80 °C until required for assays. The pancreas with attached duodenum and spleen were rapidly removed *en bloc* from each mouse.

### Tracking of hMSCs *in vivo* after infusion

hMSCs were labeled with the firefly-luciferase reporter gene (Promega, Madison, WI, USA) using the lentiviral transfection method at a MOI of 50, then the correlation of luciferase activity and cell number was analyzed by *in vitro* bioluminescence with a commercial kit (luciferase assay system; Promega). The Caliper IVIS Lumina II *in vivo* imaging system (Caliper Life Sciences, Hopkinton, MA, USA) was used to follow the distribution of MSCs over time *in vivo*. Transfected MSCs (approximately 2 × 10^6^) were infused into the mice via the tail vein at 6 h after operation, and fluorescence was assayed after intraperitoneal injection of the *in vivo* imaging substrate (VivoGlo™ Luciferin, *in vivo* grade; Promega) for up to 72 h thereafter (15 min, 30 min, 3, 6, 24 and 72 h). To further quantitatively detect the amount of hMSCs in the pancreas, CFSE-DA-labeled hMSCs were analyzed in the normal or injury pancreas 12, 24, and 72 h after injection by FACS. CM-Dil was used to detect the distribution of hMSCs in specific tissues for this experiment. In this process, cells were incubated with 2 μM CM-Dil for 15 minutes at 37 °C and for an additional 20 minutes at 4 °C, and the fluorescent staining was identified by fluorescence microscopy (DMI6000B; Leica, Wetzlar, Germany). The labeled cells were then washed three times with PBS and injected into mice via the tail vein. At 72 h after injection, mice were sacrificed. Specific tissues were removed, embedded in O.C.T. compound and stored at −80 °C until use. Frozen tissues were sectioned every 4 μm and observed with a confocal laser microscope (Carl Zeiss AG, Jena, Germany) at a magnification of ×10 and ×40.

### Real-time reverse transcription-polymerase chain reaction (RT-PCR) analysis

To analyze the expression levels of TSG-6 *in vivo* and *ex vivo*, total RNA was isolated from both lung tissue (the main organ for hMSC accumulation) and hMSCs. The lungs were removed from the mice after sacrifice, then minced into small pieces and homogenized using a motor-driven homogenizer. In addition, hMSCs, hFs and HUVECs were treated with or without 10 ng TNF-α/mL. Total RNA was then extracted from the cells or lung tissue using an RNeasy Mini Kit (Qiagen) according to the manufacturer’s directions. A Nanodrop spectrophotometer (Nanodrop Technologies, Wilmington, DE, USA) was used to measure the total RNA concentration, and the quality was also assessed by the 260/280 and 230/260 ratios. Reverse transcription was carried out with 1 μg of total RNA using PrimeScript RT Master Mix (Takara Bio Inc., Otsu, Japan). cDNA amplification was performed by real-time PCR (Light Cycler 480, Roche, Basel, Switzerland) using the GoTaq qPCR Master Mix (Promega). Beacon 7.0 Primer Express software (Applied Biosystems, Carlsbad, CA, USA) was used to design RT-PCR primers. The specific primers employed were as follows: TSG-6 forward 5′-ATA TGG CTT GAA CGA GCA GC-3; reverse 5′-GCA GCA CAG ACA TGA AAT CC-3′; 18 s forward 5′ CCT GGA TAC CGC AGC TAG GA 3′; reverse 5′ GCG GCG CAA TAC GAA TGC CC 3′. Real-time PCR reactions were incubated at 95 °C for 2 min, and amplified by 40 cycles of 95 °C for 3 s followed by 60 °C for 30 s. Relative mRNA expression levels of TSG-6 were calculated according to the 2^−ΔΔ/Cts^ method normalized to mRNA levels of 18 S.

### Histopathology

After sacrifice, pancreases were excised and preserved in 10% paraformaldehyde prior to processing and embedding. For analysis, 4-μm sections from each sample were stained with hematoxylin and eosin (H&E; Sigma-Aldrich) for histopathological evaluation of pancreatic injury using a standard procedure, or subjected to immunohistochemical staining. Each tissue section was observed by light microscopy at a magnification of ×200. Sections were evaluated from six randomly-selected fields by two separate pathologists in a blinded manner. Severity of pancreatic injury (H&E staining) was determined by employing a semi-quantitative score to assess the following three key parameters of NaT-induced SAP: (1) pancreatic edema (2) extravascular infiltration (3) acinar cell necrosis. Each parameter was evaluated on a scale from 0 to 4: (1) the degree of pancreatic edema (0 = absent; 1 = diffuse expansion of interlobar septa; 2 = diffuse expansion of interlobular septa; 3 = diffuse expansion of interacinar septa; 4 = diffuse expansion of intercellular septa), (2) extravascular infiltration (0 = absent; 1 = extravascular infiltration at 1–2 sites; 2 = 3–4 sites; 3 = 5–6 sites; 4 = ≥7 sites, and (3) necrosis (0 = absent; 1 = 1–10 necrotic cells/high-power field [HPF]; 2 = 10–20 necrotic cells/HPF; 3 = 20–30 necrotic cells/HPF; 4 = ≥30 necrotic cells/HPF). Immunohistochemistry was performed primarily to confirm the presence of neutrophils and expression of inflammatory cytokines in the pancreas. The formalin-fixed pancreas sections were deparaffinized in xylene and rehydrated through a graded ethanol series. They were then treated with 3% hydrogen peroxide for 10 min to inactivate endogenous peroxidase and washed with phosphate-buffered saline (PBS) for 5 min. After microwave antigen retrieval, sections were washed in PBS and blocked with 5% bovine serum albumin (BSA) for 1 h at room temperature. Sections were then incubated overnight at 4 °C with the following primary antibodies: MPO (1:200) (Abcam, Cambridge, UK), TNF-α (1:200) (Abcam), IL-1β (1:200) (Abcam), IL-6 (1:200) (Abcam), IL-4 (1:100) (Santa Cruz Biotechnology), IL-10 (1:100) (Santa Cruz Biotechnology), MMP-9 (1:300) (Abcam). After washing with PBS three times and then incubating with secondary antibody linked with horseradish peroxidase (DAKO; Glostrup, Denmark) for 1 h at room temperature, DAB staining was performed using a DAB peroxidase substrate kit. Nuclei were counterstained with hematoxylin, and the sections were then dehydrated through ethanol and xylene before coverslips were applied. Negative controls were processed in the same way but without the primary antibody. Semiquantitative results of immunohistochemical analysis in the pancreatic tissue were scored using the formula ∑ PI, where P represents the percentage extent of positive staining scored as 0 (0%), 1 (1–25%), 2 (26–50%), 3 (51–75%), and 4 (76–100%) and I represents the staining intensity scored as 0 (normal), 1 (weak), 2 (medium), 3 (strong). To detect acinar cell apoptosis or pyroptosis, TUNEL assay was performed using a QIA39 FragEL™ DNA Fragmentation Detection Kit with Fluorescent TdT Enzyme (Merck KGaA, Darmstadt, Germany) following the manufacturer’s instructions. At the end of the assay, six fields were randomly selected and the numbers of indicated cells in the pancreatic sections were blindly evaluated by counting the red-labelled cells under a high magnification (×63).

### Measurement of amylase, lipase, myeloperoxidase, cytokines, and chemokines

A colorimetric assay kit (BioVision, Milpitas, CA, USA) was used to measure serum amylase activity with ethylidene-pNP-G7 as the substrate. Serum lipase levels were also determined using a colorimetric assay kit (BioVision) according to the manufacturer’s protocol. The concentrations of amylase and lipase are expressed as units per liter (U/L). Neutrophil sequestration within the pancreas was quantified by measuring tissue myeloperoxidase (MPO) activity as previously described. Pancreatic tissue samples were homogenized in 20 mM phosphate buffer (pH 7.4) and centrifuged at 4 °C for 10 min at ~10,000 × *g*. Then, 50 mM phosphate buffer (pH 6.0) containing 0.5% hexadecyltrimethylammonium bromide was used to resuspend the pellet obtained. The suspension was subjected to three cycles of freezing and thawing, and further disrupted by sonication (40 seconds). Following the samples were centrifuged at 4 °C for 5 min at 10,000 × *g*, and supernatants were used for the MPO assay. Aliquots of the supernatant (50 μL) were incubated in a reaction solution consisting of 1.6 mM tetramethylbenzidine, 80 mM sodium phosphate buffer, and 0.3 mM hydrogen peroxidase. The mixture was incubated at 37 °C for 110 s, the reaction was terminated with 2 mol/L H_2_SO_4_, and the absorbance at 450 nm was recorded. The results were adjusted for protein concentration, and MPO activity was expressed as units per milligram of protein (U/mg). Measurement of cytokines (TNF-α, IL-1β, IL-6, IFN-γ) and chemokines (MCP-1, FKN) in serum or cell culture supernatant were performed with mouse-specific Quantikine ELISA Kits (R&D Systems) following the manufacturer’s instructions.

### Quantification of cell viability

For PACs apoptosis analysis *ex vivo*, cells treated with or without NaT (30 min) and concentration-dependent rhTSG-6 were preincubated with AnnexinV (Bio Basic Inc., Markham, ON, Canada) at room temperature in the dark for 15 min, followed by the addition of propidium iodide (PI, Sigma). After staining, the percentage of apoptotic cells among 10,000 cells was analyzed using flow cytometry (BD Biosciences). The Q2 region represents late apoptotic cells, and the Q4 region represents early apoptotic cells. To evaluate acinar cell viability additionally, the Cell Titer-Glo luminescent cell viability assay kit (Promega) was used to measure ATP levels that reflect cell viability. Furthermore, we also evaluated the proliferation of acinar cells using a cell counting Kit-8 (CCK-8; Dojindo, Kumamoto, Japan) according to the manufacturer’s instructions.

### Transfection of pancreatic acinar cells with CD44 siRNA

Considering the binding capacity of TSG-6 with HA-CD44 complexes and endocytosis of CD-44, a solution with 0.5% w/w of hyaluronic acid (HA, sodium hyaluronate, average molecular mass: 1.3 × 10^6^) dissolved in medium containing 1% v/v AB (HA solution) was used for preliminary incubation of NaT-treated PACs with or without rhTSG-6. Then TSG-6 and CD-44 in PACs were detected using immunofluorescence assays. To knockdown CD44 expression, PACs were transfected with CD44 siRNA or control siRNA (Santa Cruz Biotechnology) using Lipofectamine™ 2000 (Invitrogen) according to the manufacturer’s instructions. After 48 hours, CD44 knockdown was assessed using immunofluorescence assays and western blotting.

### Analysis of oxidative stress, NLRP3 inflammasome and NF-κB activation

Colorimetric assay kits (Jianchen Scientific Co. Ltd, Nanjing, China) were used to measure the activity of MDA and SOD in SAP. Briefly, MDA was determined using the thiobarbituric acid method, and the SOD assay used a tetrazolium salt for the detection of superoxide radicals generated by xanthine oxidase and hypoxanthine. *In vitro*, ROS production by NaT-treated PAC was also detected using confocal imaging with a Zeiss LSM510 and LSM710 system (Carl Zeiss AG, Jena, Germany) after loading with 5-chloromethyl-2,7-dichlorodihydrofluorescein diacetate acetyl ester (DCFH-DA, 4.5 mol/L; excitation, 488 nm; emission, 505–550 nm). Quantitative analyses were further performed by flow cytometric detection (BD Biosciences). Similar to the method previously described, real-time RT-PCR was performed again to measure mRNA transcript levels of NLRP3/ASC/Casp-1axis in pancreatic tissue. PCR conditions were as follows: 40 cycles of 95 °C for 20 s (denaturation), 55 °C for 30 s (annealing), and 60 °C for 30 s (extension). Ct values obtained were used to quantify mRNA expression. Western blot analysis of ASC, Casp-1 p20, pro-Casp-1 and β-actin in the cytoplasm, and NF-κB p65 and Histone H3 in the nucleus were performed using PAC and pancreatic tissue in the different groups. The BCA protein assay kit was employed to determine protein concentration (Pierce Biotechnology, Rockford, IL, USA). Subsequently, the extracted proteins were separated by sodium dodecyl sulfate polyacrylamide gels (SDS-PAGE; Bio-Rad, Hercules, CA, USA) and then transferred to a polyvinylidene difluoride membrane (Millipore, Billerica, MA, USA). The membrane was blocked with 5% (w/v) non-fat dried milk in Tris-buffered saline/0.05% Tween-20 (TBST) at room temperature for 1 h in a covered container and incubated overnight at 4 °C with anti-ASC (1:1000), anti-Casp-1 p20 (1:1000), anti-pro-Casp-1 (1:1000), anti-NF-κB p65 (1:400 dilution), anti-histone-H3 (1:400 dilution), or anti-β-actin (1:1000) diluted in 5% BSA. After washing, the membrane was incubated for 1 h at room temperature with secondary goat anti-rabbit antibody (1:2000) or goat anti-mouse antibody (1:2000) (Santa Cruz Biotechnology) conjugated to horseradish peroxidase. Immunoreactive proteins were visualized by chemiluminescence using the western blotting detection system (Amersham Biosciences, Little Chalfont, UK). To further evaluate NF-κB expression in the different groups, immunofluorescent staining was performed on 4-μm sections of routinely-prepared paraffin-embedded pancreatic tissues. Briefly, sections were washed three times with PBS and then blocked with 1% BSA for 30 min at room temperature. The monoclonal rabbit antibody against mouse NF-κB p65 (1:400; CST, Cell Signaling Technology Inc., Boston, MA, USA) was used as the primary antibody and anti-rabbit IgG Fab2 (1: 1,000; CST) as the secondary antibody. Finally, the preparations were washed with PBS and mounted in fluorescent mounting medium with DAPI (Invitrogen). Negative controls were processed in the same way but without the primary antibody. Each tissue section was observed under a confocal laser scanning microscope (Carl Zeiss AG) at a magnification of ×630.

### Analysis of monocyte–endothelial cell adhesion

The monocyte–endothelial cell adhesion assay was performed as described in a previous study^34^. HUVECs were grown to confluence in 6-well plates and treated with SAP serum or SAP serum + rhTSG-6 for 24 h. HUVECs were first labeled with Hoechst (Invitrogen), prior to addition of THP-1 cells labeled with CFSE (Invitrogen) for 1 h to allow adhesion. Afterwards, the medium containing monocytes was aspirated and the monolayer was gently washed with PBS three times to remove unbound monocytes. The fluorescence was measured at excitation and emission wavelengths of 496 and 516 nm under a fluorescence microscope (Leica DMI6000). The number of adherent cells was expressed as fluorescence intensity and the adhesion data are represented in terms of the fold change compared with control values. Three fields were captured per experimental condition. Individual treatments were performed in duplicate, and the entire set of experiments was repeated three times.

### Statistical analysis

All results are presented as mean values ± standard deviation (SD). Statistical analyses were performed using SPSS statistical software (IBM Corp., Armonk, NY, USA). Multiple comparisons of parametric data were performed using one-way analysis of variance (ANOVA), followed by the Student-Newman-Keuls post-hoc tests. Nonparametric data were compared using the Mann-Whitney *U-*test to identify differences between groups, and α was divided by the number of comparisons (α/comparisons) to ensure α = 0.05. A value of *P* < 0.05 was considered to be statistically significant.

## Results

### Characterization of hMSCs

The hMSCs used in this study were confirmed to have fibroblast-like morphology under the microscope, and they were adherent and showed a strong proliferative ability when cultured on plastic culture plates (Fig. S2A). In addition, they were demonstrated to have excellent multi-lineage plasticity and successfully differentiated into adipocytes, osteoblasts and chondrocytes when they were induced *in vitro* by adipogenic, osteogenic, and chondrogenic media, respectively (Fig. S2B). Flow cytometric determination of the markers on these cells showed that they were uniformly positive for CD44, CD73, CD90 and CD105, but negative for CD45 (leukocyte marker), CD34 (endothelial cell marker), CD11b (monocyte marker), CD19 (B lymphocyte marker) and HLA-DR (Fig. S2C). All the above conformed to the standard proposed by the international institute for cell therapy (ISCT), confirming that these cells were suitable for use in transplantation of the experimental animal model of SAP.

### Intravenously-administered hMSCs ameliorated severe acute pancreatitis

**Figure 1:**
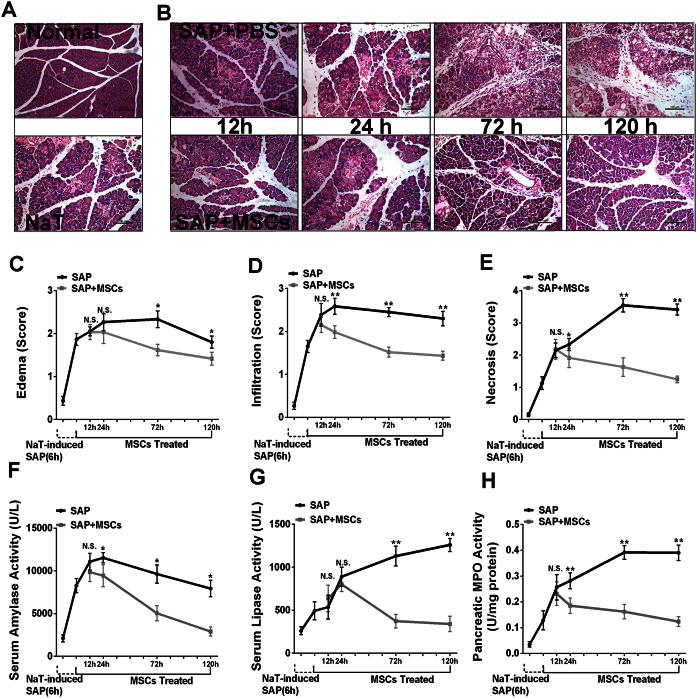
Effects of hMSCs on NaT-induced alterations of pancreatic pathology and time course of cell markers. (**A**) Representative hematoxylin/eosin (H&E) stained sections of pancreas from a control mouse demonstrate that the histological features of the pancreas showed typical normal architecture. In contrast, pancreas sections of NaT-treated mice exhibit tissue injury characterized by pancreatic edema, extravascular infiltration and acinar cell necrosis. **(B)** Pancreas sections from SAP mice at 12, 24, 72 and 120 h after receiving 2 × 10^6^ hMSCs show fewer histological alterations. Original magnification: ×200. **(C,D,E)** Histological analysis of pancreatitis severity. **(F,G,H)** Activities of amylase (U/L), lipase (U/L), and MPO (U/mg). Each value represents the mean ± standard deviation (n = 4 or 6 per group). N.S., not significant, *P < 0.05 and **P < 0.01, in comparisons between SAP control groups and SAP + hMSC groups at different time points.

Compared with the normal pancreas, histological examination of the pancreas after SAP induction (6 h) showed tissue injury characterized by markedly edema and some necrosis of acinar cells (Fig. 1A). In the SAP group without hMSC treatment, the pancreatic tissue developed severe damage characterized by a large number of necrotic cells, vacuolization and inflammatory cell infiltration. In contrast, extravascular infiltration and necrosis of pancreatic acinar cells were significantly reduced by treatment with intravenous hMSCs compared with the control group at 24 h (*P* < 0.01 and *P* < 0.05), 72 h (*P* < 0.01) and 120 h (*P* < 0.01), and the degree of pancreatic edema was also improved at 72 h (*P* < 0.05) and 120 h (*P* < 0.05) after the same treatment (Fig. 1B,C,D,E). The analysis revealed that hMSC-treatment markedly decreased serum amylase levels at 24, 72 and 120 h (*P* < 0.05). Similar results were observed in serum lipase activity which also showed a significant reduction (Fig. 1F,G). MPO is an enzyme that is found predominantly in the azurophilic granules of polymorphonuclear leukocytes (PMN). Therefore, tissue MPO activity is commonly utilized to assess tissue PMN accumulation in inflamed tissues. As expected, pancreatic MPO activity was markedly increased in SAP animals after NaT induction, and was also suppressed significantly after hMSCs treatment (Fig. 1H).

### Intravenously-administered hMSCs did not accumulate in the injured pancreas

**Figure 2:**
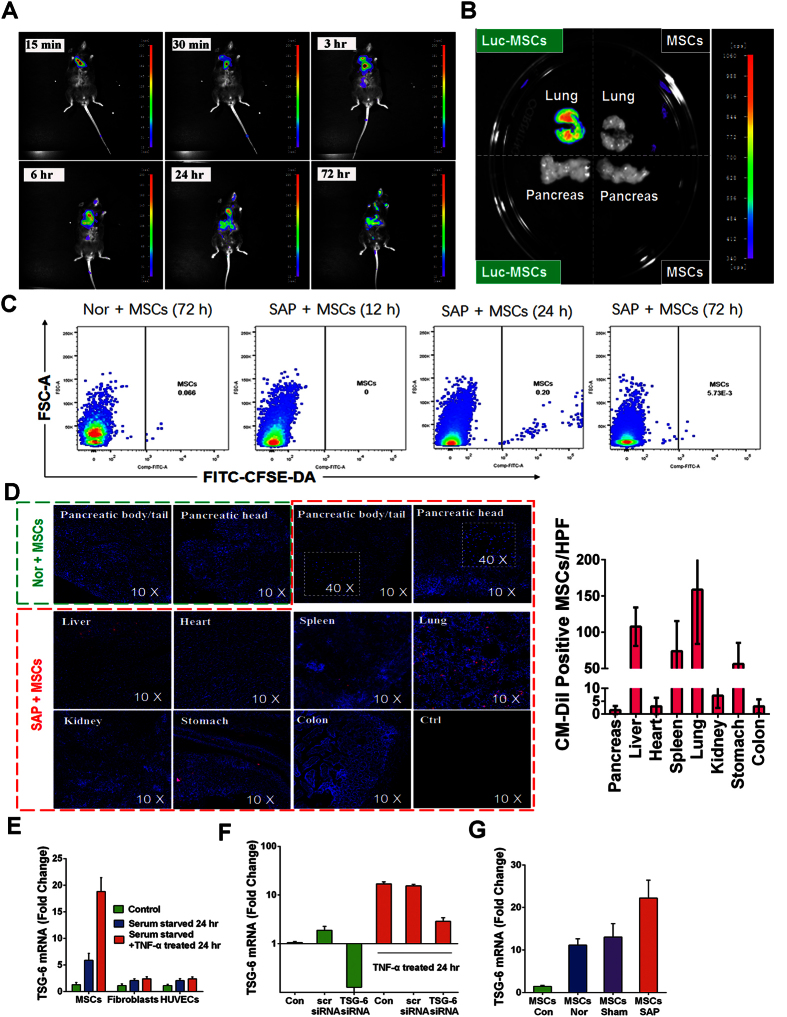
Tracking the distribution of human mesenchymal stem cells infused into mice via the tail vein, and activation of hMSCs or other cells to express TSG-6. **(A)** The results of *in vivo* imaging show that Luc-hMSCs injected via the tail vein first spread in the lungs and gradually accumulated in the liver and spleen. There was no apparent signal in the pancreas throughout the entire experimental period. **(B)** An intense signaling was only found in the lung but not the pancreas at 72 h after the i.v. injection of Luc-hMSCs. **(C)** Representative Dot Plots of CFSE-DA-positive hMSC frequency at different time-points after the injection. All frequencies are referred to viable cells. **(D)** With the help of a confocal laser microscope, CM-Dil-labeled cells (Red) in different organs of a mouse were detected 72 h after hMSC injection and were analyzed in eight randomly selected fields from four mice at 10× or 40× magnification for each group. **(E)** Real-time RT-PCR assays for TSG-6 in hMSCs, human fibroblasts, and human umbilical vein endothelial cells (HUVECs) incubated in serum-free medium with or without 10 ng/mL TNF-α for 24 h. Data are presented as mean ± standard deviation; n = 5. **(F)** Real-time RT-PCR assays of TSG-6 in control hMSCs (Con), hMSCs transfected with a scrambled siRNA (scr siRNA), or hMSCs transduced with TSG-6 siRNA (TSG-6 siRNA). Cells were incubated in serum-free medium with or without 10 ng/mL TNF-α for 24 h. Data are presented as mean ± standard deviation; n = 3. **(G)** Real-time RT-PCR assays for human TSG-6 in the lungs of normal mice (hMSCs nor), sham-operated mice (hMSCs sham), and SAP mice (hMSCs SAP) 24 h after infusion of approximately 2 × 10^6^ hMSCs. hMSCs control refers to the normal growth state of these cells without any treatment. Data are presented as mean ± standard deviation; n = 3 for normal or sham-operation mice; n = 5 for SAP mice.

Firefly-luciferase, CFSE-DA, or CM-Dil-labeled cells maintained their proliferative ability and fibroblast-like shape *in vitro*, indicating the stability of hMSCs after the labeling process. An *in vivo* imaging system was utilized to follow the distribution of cells after injection into the SAP mouse via the tail vein. According to the intensity of the captured fluorescence signal, Luc-hMSCs began to spread in the lung 30 min after injection. The signal began to markedly accumulate in the liver 6 h after transplantation and gradually reached the spleen, but primarily accumulated in the lungs and liver 24 h after injection. There was no apparent signal in the injured pancreas throughout the entire period (Fig. 2A). In addition, we detect highly visible signal in the lung at 72 h after the Luc-hMSCs injection but not for the injection of hMSCs without firefly-luciferase reporter gene. More importantly, in the pancreas, there were no any signals after the i.v. transplantation of hMSCs with or without firefly-luciferase reporter gene (Fig. 2B). Healthy and AP mice were injected intravenously with CFSE-DA-labeled hMSCs. Pancreases were collected after hMSCs injection from all mice and analyzed for CFSE-DA fluorescence parameters by FACS. No significant CFSE-DA-positive cells were detected in the pancreas of normal and AP mice, whereas CFSE-DA-labeled cells appeared in the inflamed pancreas of MSC-treated AP mice at 24 h after injection with a very low frequency (0.20%) (Fig. 2C). Immunofluorescence analysis also revealed no significant engraftment of CM-Dil-labeled hMSCs in the injured pancreas (<5/Field) 72 h after transplantation, but showed that the hMSCs were still primarily trapped in the lungs (>150/Field). No cell-specific red fluorescence was detected in sections from SAP mice that had not received the CM-Dil-labeled hMSCs (Fig. 2D). Therefore, the therapeutic effects of the hMSCs could not be explained by the i.v. administered cells engrafting in the pancreas.

### hMSCs *in vitro* and *in vivo* are activated to secrete TSG-6

As a multifunctional and powerful anti-inflammatory protein, the therapeutic effects of TSG-6 have been confirmed in many animal models. TSG-6 was originally identified as a cDNA derived from human fibroblasts treated with TNF-α, but it has since been demonstrated that TSG-6 can also be secreted by different types of cell in an inflammatory environment. In this study, hMSCs, hFs and HUVECs were treated by serum starvation or incubated with hTNF-α for 24 h, and gene expression was then assayed by real-time RT-PCR. The results showed that human TSG-6 mRNA in hMSCs was increased approximately 6-fold or 19-fold respectively after serum starvation or hTNF-α incubation, which far exceeded the response of hFs or HUVECs (Fig. 2E). Additionally, we also knocked down the expression of TSG-6 in hMSCs by transient transfection with a TSG-6 siRNA and found that the effects of TNF-α were also abrogated. Real time RT-PCR assays demonstrated that the knockdown efficiency was approximately 87% (Fig. 2F). To further explore the expression of TSG-6 *in vivo* in the SAP environment after i.v. infusion of hMSCs, the transcript for TSG-6 in the lung was also assayed by real-time RT-PCR. As expected, the results showed that TSG-6 mRNA levels in the SAP group were remarkably higher than in the sham group or the normal group (Fig. 2G).

### Both hMSCs and rhTSG-6 suppressed pancreatic injury in mice with SAP

**Figure 3:**
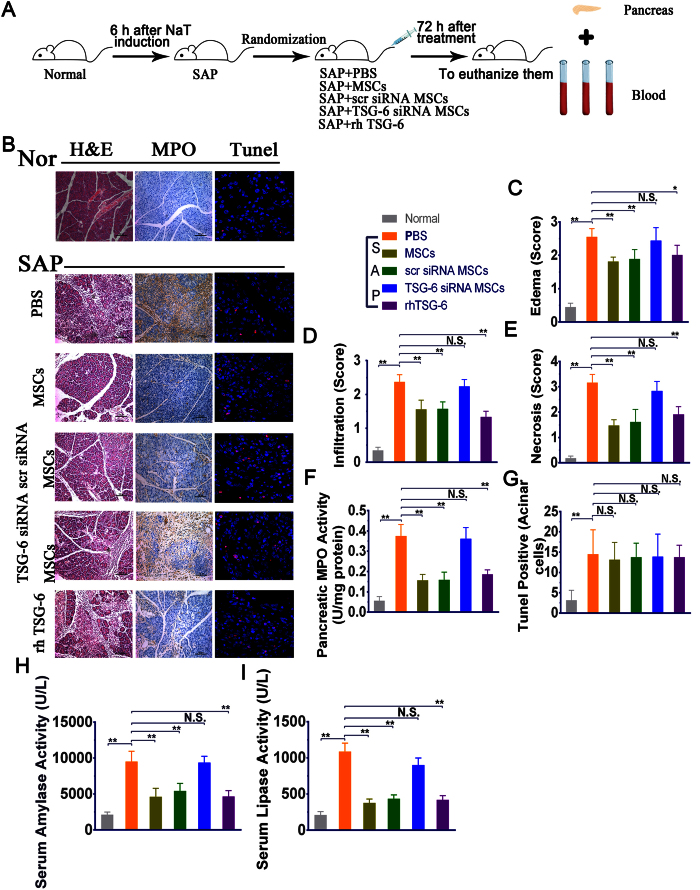
Effects of hMSCs, scr siRNA hMSCs, TSG-6 siRNA hMSCs and rhTSG-6 on NaT-induced alterations of pancreatic pathology and cell markers. **(A)** In a separate study, mice initially received NaT by retrograde biliary and pancreatic duct infusion, followed by intravenous injection 6 hours later with PBS, hMSCs, scr siRNA hMSCs, TSG-6 siRNA hMSCs, and rhTSG-6. Mice were then sacrificed 3 days later to evaluate the cell migration and therapeutic effects. **(B)** Representative H&E, immunohistochemical staining of MPO and TUNEL staining in pancreatic tissue sections in each group. H&E staining evaluation revealed the effects of each treatment on pancreatic injury and inflammation, while immunohistochemical evaluation of MPO revealed the numbers of polymorphonuclear leukocytes. Original magnification: ×200. TUNEL staining revealed the number of cell s undergoing apoptosis or pyroptosis. Original magnification: ×630. **(C–G)** The histology scores of H&E, activity of MPO (U/mg) and number of TUNEL-positive cells after operation with i.v. administration of hMSCs, scr siRNA hMSCs, TSG-6 siRNA hMSCs and rhTSG-6. **(H,I)** The activities of amylase (U/L) and lipase (U/L) after operation with i.v. administered hMSCs, scr siRNA hMSCs, TSG-6 siRNA hMSCs and rhTSG-6. Each value represents the mean ± standard deviation (n = 8 or 12 per group). N.S., not significant, *P < 0.05 and **P < 0.01, compared with SAP + PBS group.

To further evaluate the role of TSG-6 secreted by hMSCs in the improvement and recovery of SAP, PBS, hMSCs transduced with or without TSG-6 siRNA and rhTSG-6 were individually infused into the SAP mice via the tail vein (Fig. 3A). Compared with PBS-treated controls, the degree of pancreatic edema, extravascular infiltration and necrosis of pancreatic acinar cells were all significantly reduced by i.v. administered hMSCs and hMSCs with scrambled siRNA (*P* < 0.01), but not by hMSCs transduced with TSG-6 siRNA. In addition, treatment with rhTSG-6 alone also resulted in significant therapeutic effects similar to that observed with hMSCs (*P* < 0.05 or *P* < 0.01) (Fig. 3B,C,D,E). Furthermore, MPO activity was significantly reduced at 72 h after i.v. administration of hMSCs or hMSCs with scrambled siRNA (*P* < 0.05 or *P* < 0.01). The beneficial effects of hMSCs were obviously negated by knockdown of the TSG-6 gene prior to infusion of the cells, however, infusion of 30 μg rhTSG-6 partially duplicated the effects of hMSCs and the effect was consistent with its known anti-inflammatory effects (Fig. 3B,F). Apart from cellular necrosis, apoptosis and pyroptosis are also marked features in the pathological conditions of SAP. To further examine whether they were also mediated by treatment with hMSCs or rhTSG-6, TUNEL staining was performed on the pancreatic sections. Our results showed that cell apoptosis and pyroptosis are present in NaT-induced SAP, however, there are no statistically-significant differences between any of the treatment groups (Fig. 3B,G). Serum amylase and lipase are most commonly considered to be biochemical markers of pancreatic disease, especially SAP. Therefore, we estimated the severity of SAP by detecting and comparing the levels of enzyme production 72 h after different treatments. Compared to PBS-injected controls, both hMSCs and hMSCs with scrambled siRNA significantly decreased the levels of serum amylase and serum lipase, as well as the administration of rhTSG-6, while hMSCs with TSG-6 knockdown had no effect (*P* < 0.01). (Fig. 3H,I).

### Both hMSCs and rhTSG-6 reduced the pancreatic and systemic inflammatory response.

**Figure 4:**
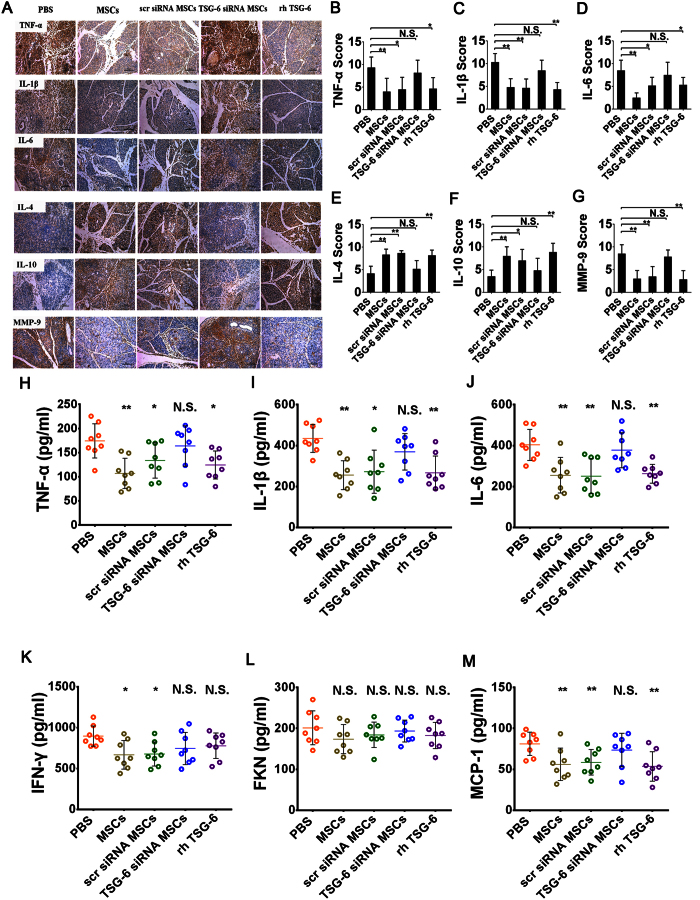
Effects of hMSCs, scr siRNA hMSCs, TSG-6 siRNA hMSCs and rhTSG-6 on the production of inflammatory cytokines and chemokines. **(A)** Immunohistochemical evaluation revealed the effects of each treatment on the expression level of TNF-α, IL-1β, IL-6, IL-4, IL-10, and MMP-9 in the pancreas of SAP mice. Original magnification: ×200. **(B–G)** The immunohistochemical scores of TNF-α, IL-1β, IL-6 and MMP-9 after operation were reduced, but IL-10 and IL-4 elevated, with i.v. administration of hMSCs, scr siRNA hMSCs and rhTSG-6 apart from TSG-6 siRNA hMSCs. Elisa assays for serum inflammatory cytokines **(H)** TNF-α, **(I)** IL-1β and **(J)** IL-6, **(K)** IFN-γ, and chemokines **(L)** FKN and **(M)** MCP-1. Each value represents the mean ± standard deviation (n = 8 or 12 per group). N.S., not significant, *P < 0.05 and **P < 0.01, compared with the SAP + PBS group.

Semiquantitative results of our immunohistochemical analysis showed that treatment of hMSCs or hMSCs transduced with a scrambled siRNA can significantly inhibit the activation and release of these pro-inflammatory cytokines (TNF-α, IL-1β and IL-6) and play a promotional role in the production of anti-inflammatory cytokines (IL-4 and IL-10) compared with PBS (*P* < 0.05 or *P* < 0.01), but the same effect was not observed in hMSCs transduced with TSG-6 siRNA despite similar numbers. Furthermore, the beneficial effects of hMSCs simulated by rhTSG-6 were consistent (*P* < 0.05 or *P* < 0.01) (Fig. 4A,B,C,D,E,F). Matrix metalloproteinases (MMPs), especially MMP-9, are generated in the inflammatory reaction process of SAP. Not only is one of their effects similar to that of trypsin in that they digest pancreatic tissues and activate other enzymes, but also their expression level can reflect the number of infiltrating granulocytes and monocytes in the injured pancreas. As expected, the level of MMP-9 was significantly reduced after treatment of hMSCs, either untreated, treated with scrambled siRNA or with rhTSG-6 (*P* < 0.01). In addition, there was no significant difference between the TSG-6 siRNA group and the PBS group. (Fig. 4A,G). To further evaluate the system inflammatory changes after treatments, levels of inflammatory cytokines and chemokines were also measured in serum samples collected from animals. As shown in Fig. 4H,I,J,K, the levels of proinflammatory cytokines (TNF-α, IL-1β, IL-6 and IFN-γ) were all reduced significantly after treatment with hMSCs or hMSCs with scrambled siRNA compared with the PBS-infused controls (*P* < 0.01 or *P* < 0.05). In addition, infusion of rhTSG-6 also exhibited anti-inflammatory effects, markedly decreasing the levels of TNF-α, IL-1β, and IL-6 (*P* < 0.01or *P* < 0.05), but not IFN-γ. hMSCs transfected with TSG-6 siRNA, however, did not show reduced levels of all proinflammatory cytokines. Furthermore, i.v. administration of hMSCs, hMSCs with scrambled siRNA or rhTSG-6 all significantly attenuated the increase of serum MCP-1, a vital chemokine in the pathogenesis of pancreatitis (*P* < 0.01), while hMSCs with TSG-6 knockdown had no effect. The levels of another chemokine, FKN, were also measured after treatment, but we found no statistically significant differences between any of the groups by post-hoc analysis (Fig. 4L,M).

### rhTSG-6 improved the viability of pancreatic acinar cells *in vitro*

**Figure 5:**
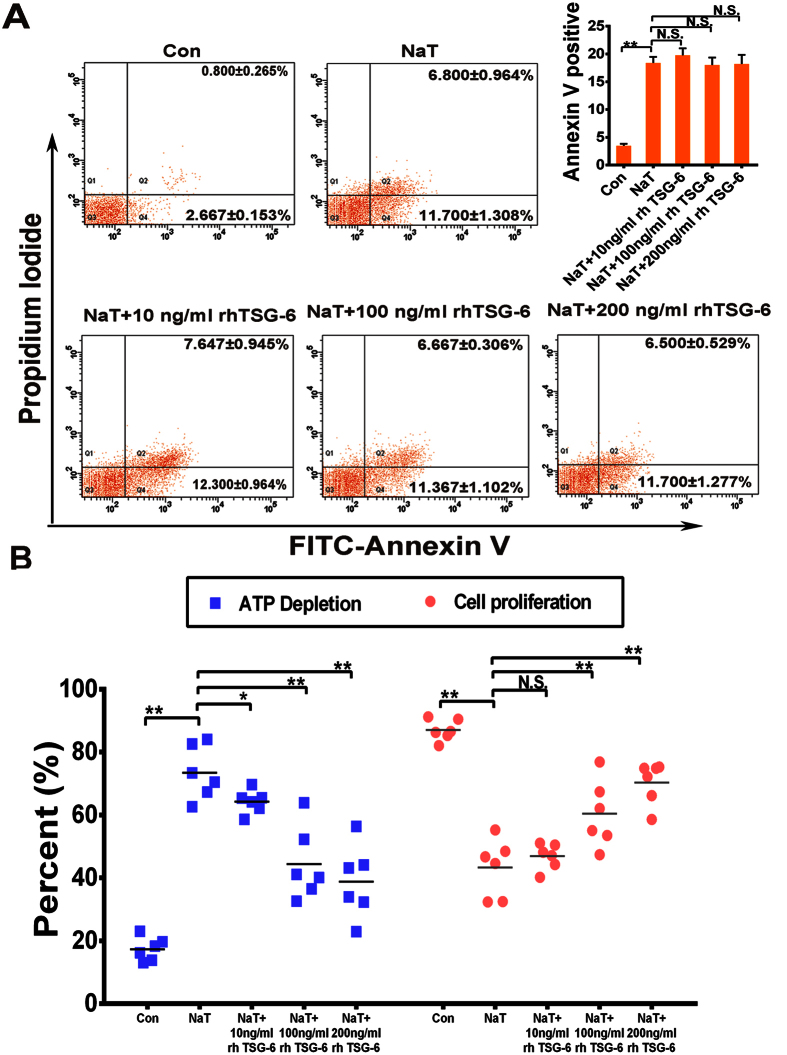
rhTSG-6 improved the activity of NaT-treated pancreatic acinar cells. **(A)** *In vitro*, acinar cells in different group were collected and stained with annexin V-FITC/propidiumiodide and detected by flow cytometry. The right panel indicates the apoptotic cells. Figures are representative of three independent experiments. Each value represents the mean ± standard deviation. N.S., not significant and **P < 0.01, compared with NaT-treated groups. **(B)** Acinar cell necrosis was reflected by analysis of ATP levels. rhTSG-6 significantly decreased the depletion of ATP in injured acinar cells in a dose-dependent manner, **(C)** and increased proliferation in a dose-dependent manner. Each value represents the mean ± standard deviation (n = 6 per group). N.S., not significant, *P < 0.05 and **P < 0.01, compared with NaT-treated groups.

PACs are the functional unit for external secretion by the pancreas, and account for 80% of pancreatic tissue. Based on the above evidence, we then determined whether rhTSG-6 contributes to pancreatic acinar cell survival *in vitro*. The viability of PACs incubated with or without NaT (375 nmol/mL) and rhTSG-6 at different doses (0, 10, 100, and 200 ng/mL) was analyzed by flow cytometry after staining with annexin V-FITC/propidium iodide (PI), Cell Titer-Glo Luminescent Cell Viability Assay kit and Cell Counting Kit-8. Similar *in vivo* results suggested that rhTSG-6 significantly improved the survival of PACs (*P* < 0.01 or *P* < 0.05), but did not affect apoptosis (Fig. 5A,B).

### The beneficial effects of hMSCs were dependent on its secreted TSG-6 in SAP in a mechanism involving oxidative stress, NLRP3 inflammasome and NF- kB signaling

**Figure 6:**
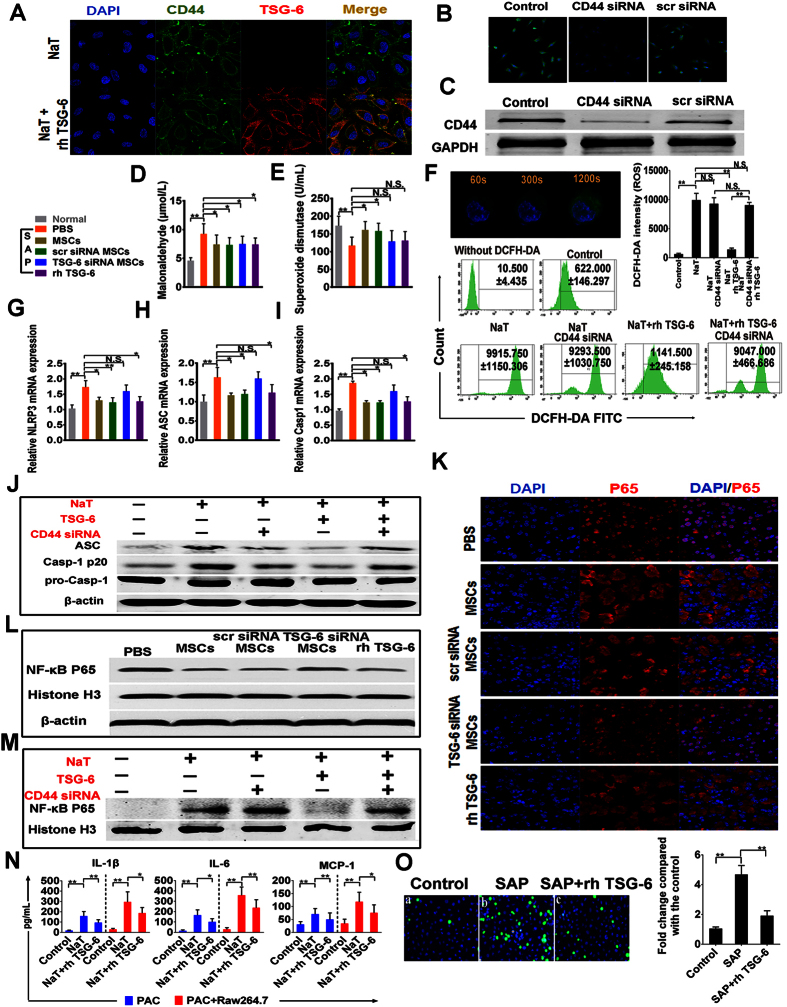
The beneficial effects of hMSCs were dependent on its secreted TSG-6 in SAP in a mechanism involving oxidative stress, NLRP3 inflammasome and NF- κB signaling. **(A)** Representative immunofluorescence co-staining of TSG-6 (red) and CD-44 (green) in NaT treated-PACs. The nuclei were visualized using DAPI (blue). Figures are representative of three independent experiments. Magnification ×630. **(B,C)** Representative immunofluorescence images of CD44 (green) in control PACs, CD44-siRNA-PACs, and scr-siRNA-PACs. Nuclei are stained with DAPI (blue). Magnification ×400. Western blot assay was also performed. Figures are representative of three independent experiments. **(D)** Evaluation of MDA and **(E)** SOD in serum. The levels of MDA (μmol/L) and SOD (U/mL) were evaluated in each group, (n = 8 or 12). **(F)** Transmitted light and DCFH-DA whole cell fluorescence (green) images of murine cells showing increases in ROS induced by NaT. In addition, quantitative detection in each group were detected via flow cytometry analysis. Evaluation of mRNA transcript levels of **(G)** NLRP3, **(H)** ASC, and **(I)** Casp-1for pancreatic tissue in each group. **(J)** Western blot analysis of ASC, Casp-1 20 subunit, pro-Casp-1 in PACs. β-actin were used as the internal references. **(K)** Representative pancreatic sections of immunofluorescent staining are shown for cytoplasmic and nuclear distribution of NF-κB. Original magnification: ×630. **(L,M)** Western blot analysis of NF-κB p65 in the nucleus of pancreatic acinar cells. Histone H3 and β-actin were used as the internal references for nuclear and whole cell proteins, respectively. **(N)** The increases of IL-1β, IL-6, and MCP-1 in culture supernatant of acinar cells incubated with or without macrophages (Raw264.7) stimulated by NaT were significantly reduced with 100 ng rhTSG-6. **(O)** Confluent monolayers of HUVECs labeled with Hoechst were grown on six-well plates and stimulated with SAP serum or SAP serum +100 ng rhTSG-6 for 24 h. CFSE fluorescent intensity of adherent THP-1 cell was measured and the final results were expressed as the fold induction of CFSE fluorescence compared with controls. Each value represents the mean ± standard deviation. N.S., not significant, *P < 0.05, **P < 0.01.

CD44 is the major cell surface receptor for hyaluronan (HA), and TSG-6 has been shown to exert its anti-inflammatory effects by mediating HA-CD44 interaction. In addition, a previous study demonstrated that TSG-6 attenuates zymosan-induced mouse peritonitis by decreasing TLR2/NF-κB signaling, dependent on the expression of CD44 on macrophages. To identify the role of CD44 and the relationship between CD44 and TSG-6 in modulating the activities of NaT-treated PACS, we repeated some of the above reported experiments. As shown in Fig. 6A, TSG-6 also bound HA and attached in the cell membrane, an effect which was depend on CD44-mediated endocytosis after preliminary incubation of HA. Furthermore, the efficiency of CD44 knockdown in PACs was confirmed using immunofluorescence and western blotting (Fig. 6B,C). In order to verify the potential of hMSCs and TSG-6 to reduce oxidative stress during SAP, both MDA and SOD were measured in the murine serum collected from each group. MDA activity was significantly reduced by hMSCs and rhTSG-6 compared with the PBS-treated group, (*P* < 0.05), and additionally only hMSCs were able to significantly increase the SOD level (*P* < 0.05) (Fig. 6D,E). *In vitro*, we used the molecular probe DCFH-DA to detect ROS production in PACs induced by NaT. Fluorescence intensity reflecting the ROS production in one acinar cell (n = 8) was detected under confocal microscopy, and our results suggested that NaT induced a generalized, sustained increase in ROS activity with increasing reaction time. All increases of ROS production ceased after 20 minutes’ reaction time, and further quantitative detection in each group was performed by flow cytometry analysis at this time point. Interestingly, while CD-44-knockdown alone in NaT-treated PACs had no influence on ROS production, on the other hand TSG-6 significantly inhibited ROS production in a CD44-dependent manner (*P* < 0.01) (Fig. 6F). The NLRP3 inflammasome is an intracellular multiprotein complex triggering inflammatory responses. The mRNA expression levels of components of the NLRP3/ASC/Caspase-1 axis were all remarkably increased in the progression of SAP. As expected, treatment with hMSCs or rhTSG-6 significantly reduced their expression levels compared with the PBS-treated group (*P* < 0.05) (Fig. 6F,G,H,I). Cytoplasmic proteins, extracted from PACs in different groups, were also used to detect the expression of ASC, pro-Casp-1, and Casp-1 p20, subunits of activated caspase-1, used as one of the markers of NLRP3 inflammasome activation. Compared to the reference β-actin, the expression of pro-Casp-1 in PACs induced by NaT showed no difference between the different groups but both Casp-1 p20 and ASC expression were inhibited by rhTSG-6. However, the presence of rhTSG-6 had less effect on reducing the expression of Casp-1 p20 and ASC when CD-44 was knocked down in PACs compared with non-transfected control cells (Fig. 6J). The NF-κB pathway in acinar cells plays a vital and initial role in the induction of proinflammatory cytokines and chemokines, which then recruit a large number of inflammatory and immune cells into the damaged pancreatic tissue during SAP. In order to determine whether TSG-6 affects NF-κB activity, we examined NF-kB p65 localization and nuclear translocation via western blot and immunofluorescence detection. Immunofluorescence detection showed that hMSCs, hMSCs transfected with scr siRNA, and rhTSG-6 all remarkably inhibited nuclear translocation of NF-κB, but that hMSCs with TSG-6 knockdown showed only a weak inhibitory effect (Fig. 6K). In addition, western blot analysis of NF-κB p65, histone H3 in the nucleus and β-actin in the pancreatic tissue revealed the same tendency (Fig. 6L). Furthermore, nuclear NF-κB p65 expression in NaT-induced acinar cells was also significantly suppressed by rhTSG-6 *in vitro*. However, rhTSG-6 had little effect on CD44-siRNA-transfected acinar cells compared to non-transfected control cells (Fig. 6M). To further explore cell damage-related inflammation, we also measured levels of the proinflammatory mediator IL-1β, IL-6 and the chemokine MCP-1 in the culture supernatant of acinar cells and of acinar cells cocultured with macrophages. As shown in Fig. 6N, the increase of IL-1β, IL-6, and MCP-1 induced by NaT were significantly abrogated by treatment with rhTSG-6 (*P* < 0.01 or *P* < 0.05). Release and activation of proinflammatory mediators and chemokines can recruit a large number of inflammatory cells such as monocytes/macrophages into the injured pancreatic tissue, and in turn these inflammatory cells release a large number of inflammatory factors. Therefore, we used CFSE-DA-labeled monocytes to evaluate the effect of rhTSG-6 on adhesion of monocytes to HUVECs. As expected, administration of SAP serum for 24 h markedly increased adhesion of THP-1 cells compared with the control group (20 μL SAP serum increased adhesion 4.46-fold), and treatment with rhTSG-6 also significantly blocked the adhesion of THP-1 cells (*P* < 0.01) (Fig. 6O).

## Discussion

To the best of our current knowledge, development of SAP can be considered as a progression from an initial injury of the exocrine pancreatic tissue, which then progresses to local and systemic inflammatory responses^35^. The present study demonstrates that i.v. administration of hMSCs ameliorates NaT-induced SAP in the absence of evidence of significant engraftment of hMSCs. Our findings also confirm that the multifunctional anti-inflammatory protein TSG-6 is one of the beneficial factors secreted by hMSCs in response to the inflammatory environment, and show that TSG-6 limits the severity of pancreatitis by protecting acinar cells from oxidative stress, NLRP3 inflammasome activation, NF-κB signaling, and excessive inflammatory response associated with CD-44. We believe that TSG-6 is both necessary and sufficient to suppress inflammation and injury in the pancreas, in that hMSCs with TSG-6 mRNA knockdown lost most of their valuable effects while i.v. administered rhTSG-6 was able to largely duplicate the effects observed with hMSC treatment. These results are consistent with previous findings showing that i.v. administered hMSCs can produce preventative and therapeutic benefits without significant engrafting into injured tissues and demonstrating that they act primarily through upregulation of TSG-6 expression^27^,^28^,^29^,^36^,^37^,^38^,^39^,^40^,^41^,^42^,^43^. Our findings further explain the precise mechanism of action of hMSCs in SAP.

In an animal model of NaT infusion, the findings are highly consistent with human disease in regard to the pathophysiological processes of SAP, being characterized by pancreatic inflammation, hemorrhage and necrosis, and marked increases in serum amylase, lipase, and immune cytokine levels. In the study, it’s usually 6 h after NaT infusion that pancreatic injury/necrosis get serious, which indicates that NaT-induced AP has a more rapid progress in the mouse^31^,^32^. Not only suppressing inflammation and repairing injury, hMSCs also remarkably reduced the increase of serum amylase and lipase associated with SAP in our study. However, significant effects primarily appeared at 72 and 120 h after treatment with hMSCs, but were not apparent at 12 h. We concluded that hMSCs are potentially capable of restricting pancreatic injury produced during SAP by restoring the fine structure and maintaining the inherent physiological function of acinar cells. Furthermore, the timely reduction of pancreatic and systemic inflammation after hMSC treatment may also offer vital protection against pancreatic damage and therefore prevent the development of pancreatitis, which can cause perpetuation of an especially vicious circle. Nevertheless, the apparent onset of the beneficial effects of hMSCs may also require a certain threshold stimulus and response time^44^.

Despite significant recovery of SAP after hMSC treatment, we found no evidence of significant engraftment of hMSCs in the pancreas, and our experimental results showed that the multifunctional anti-inflammatory protein, TSG-6, accounted for the vital effects of i.v.-infused hMSCs. Accumulating evidence has demonstrated that death of acinar cells and an excessive inflammatory response are the key pathologic responses of pancreatitis and the leading cause of severe complications^4^. The initial injury in pancreatitis is characteristically sterile and results in acinar cell necrosis, which leads to the release of intracellular contents from damaged cells into the extracellular space. These then serve as damage-associated molecular patterns (DAMPs) that link local tissue damage to the inflammatory response. Above all, the sterile inflammatory response, especially the induction and activation of proinflammatory mediators and chemokines, and recruitment of immune cells, is the key determinant of further pancreatic parenchymal cell injury, remote organ injury, and disease resolution in experimental models and in human pancreatitis^45^. In our present study, knockdown of TSG-6 expression in hMSCs significantly negated the improvements in inflammatory response, pancreatic tissue injury, and levels of enzyme production generated by i.v. infusions of hMSCs. In addition, i.v. infusions of 30 μg rhTSG-6 remarkably reproduced similar role to those of the hMSCs in reducing inflammatory responses and pancreatic tissue injury. However, our study suggested that both hMSCs and TSG-6 have little effect on pancreatic acinar cell apoptosis in NaT-induced SAP. Considering the multifactorial features of acute pancreatitis, we were unable to conclude that TSG-6 or hMSCs definitely have no effect on apoptosis or pyroptosis in other diseases. In addition, some studies have shown that mild acute pancreatitis (MAP) is associated with extensive apoptotic acinar cell death, whereas SAP such as in our study involves acinar cell necrosis with little apoptosis^46^. It has even been hypothesized that apoptosis might actually represent a favorable response in acute pancreatitis^47^. TNF-α and IL-1β are key effector cytokines in the innate immune response to sterile pancreatic damage^48^, and the IL-6 they induce is a principal cytokine mediator proposed as a marker for predicting the severity of pancreatitis^49^. A previous study also showed that blockade of IL-6 can relieve experimental acute pancreatitis *in vivo*^47^. TSG-6 expression, in turn, can be largely upregulated in hMSCs following stimulation by these proinflammatory cytokines. Beyond that, increasing the activity of anti-inflammatory cytokines IL-4 and IL-10 and decreasing the activity of MMP-9 and MPO in damaged pancreatic tissue also help promote recovery of pancreatic injury. The increase of serum proinflammatory mediators (TNF-α, IL-1β, IL-6, IFN-γ) and chemokines (MCP-1, FKN) usually contribute to further pancreatic injury and remote organ injury^50^,^51^. Although we were unable to detect a significant decrease in the level of serum IFN-γ after rhTSG-6 treatment or in serum FKN level after any of the treatments, we believe this is because TSG-6 only partly mediates the immunosuppressive effects of hMSCs, and because serum FKN levels reach a peak at a relatively early time after SAP induction^52^. Amylase and lipase, as clinical markers of pancreatic injury, also aggravate ductal permeability as well as increasing release of intracellular contents from necrotic acinar cells, but are not thought to result in further injury^53^. In view of the complexity of *in vivo* conditions in a SAP animal model, the anti-inflammatory effect of TSG-6 in pancreatitis was also confirmed with a series of *ex vivo* investigations. Our findings collectively revealed that TSG-6 plays a key role in the many therapeutic and preventative benefits of hMSCs.

The mechanism underlying the effect of TSG-6 in SAP still needs be explored. Previous studies have shown that excessive oxidative stress implies the existence of early inflammation in the pancreas. In effect, pathological oxidative stress can directly attack the inherent defenses of the pancreas and enhance acinar cell sensitivity to the inflammatory response^54^,^55^. Recent findings that elimination of ROS by antioxidants suppressed activation of the NLRP3 inflammasome and the resultant maturation of IL-1β have shed new light on understanding the roles of antioxidants in the pathogenesis of inflammation-related diseases^56^. Our study confirmed that TSG-6 secreted from hMSCs may also exert an inhibitory effect on the increase of ROS production in pathological pancreatic conditions, and hence limit the persistent inflammation, especially in the early period. As an important component of the innate immune system, the NLRP3 inflammasome could be activated by endogenous molecules known as damage-associated molecular patterns (DAMPs), such as DNA, adenosine triphosphate (ATP), high mobility group box 1 (HMGB1), and nucleotide binding oligomerization domain 2 (NOD2) which are all associated with inflammation during the development of pancreatitis. Furthermore, components of the inflammasome, specifically ASC, caspase-1, and NLRP3, are required for the development of inflammation in acute pancreatitis^8^,^57^,^58^. Taken together with our results, these findings suggest that TSG-6 can act as an antagonist of the NLRP3 inflammasome, resulting in attenuation of cellular inflammatory processes and consequent relief of pancreatic injury during SAP. Previous studies revealed that NF-κB is responsible for regulating the transcription of a wide range of genes involved in inflammation and cell death in the development and progression of SAP, and the inhibition of NF-κB activation also diminishes the inflammatory response and limits the severity of pancreatitis. In light of these findings, development of NF-κB inhibition strategies to treat pancreatitis has aroused much interest^59^,^60^,^61^. As expected, the present study shows that hMSCs, hMSCs with scrambled siRNA, and rhTSG-6 all have an inhibitory effect on NF-κB activation, while hMSCs with TSG-6 knockdown show only a very weak effect. Both NF-κB and NLRP3 inflammasome activation can be induced by the production of ROS while it is accompanied by a series of other pathological events such as the increase of Ca^2+^ and mitogen-activated protein kinase (MAPK) activation^62^. More importantly, we found that the beneficial effects of TSG-6 were dependent on CD44 expression by acinar cells and these results are consistent with previous observations^28^,^42^,^63^. Our data also suggest that rhTSG-6 may protect vessels under conditions of pancreatitis from monocyte adhesion by suppressing the inflammatory response. We conclude that the multifunctional anti-inflammatory protein TSG-6 secreted by hMSCs suppresses NaT-induced SAP by mediating oxidative stress, inhibiting NLRP3 inflammasome activation and decreasing NF-κB signaling in acinar cells, thereby largely reducing inflammation and cell death.

TSG-6 was originally identified as a cDNA derived from TNF-α-treated human fibroblasts, and its corresponding expressive protein (TNAIP6) is mainly composed of a contiguous link module and complement subcomponents C1r/C1s-Uegf-BMP-1 forming the CUB module. It has been confirmed that TSG-6 is produced in inflammatory processes such as rheumatoid arthritis and in inflammation-like processes such as ovulation and cervical ripening, where its expression is probably induced by a variety of cytokines^26^. Our finding also suggested the absolute importance of secreted TSG-6 for hMSCs to produce valuable effects in SAP. Even so, our results do not rule out the possibility that hMSCs secrete other beneficial factors in addition to TSG-6. In the microenvironment of non-infectious inflammation, hMSCs can interact with immune or inflammatory cells and produce a large number of soluble cytokines such as TSG-6, hepatocyte growth factor, transforming growth factor (TGF)-β, prostaglandin (PG)E2, IL-4, IL-10, IL-1 receptor antagonist, inducible NO synthase, indoleamine 2,3-dioxygenase, galectin-1 and human leukocyte antigen (HLA)-G^64^. Additional experiments are required to determine the relative contribution of each of these factors to the beneficial effects of hMSCs in the pancreas. In addition, we also cannot exclude the possibility that a small number of hMSCs present in the damaged pancreatic tissue may also have contributed to the therapeutic effects^20^. However, TSG-6 plays a key role in most beneficial effects of hMSCs.

It is necessary to recognize certain limitations in our present study. One limitation is that the study did not explicitly reveal that TSG-6 is a key factor in improving recovery of pancreatic function following pancreatitis after treatment with hMSCs, especially β-cell function^65^. Furthermore, since SAP is always accompanied by remote organ damage in addition to pancreatic injury, whether hMSCs will also have beneficial effects on these remote organs under this disease condition remains undefined. Therefore, it will be necessary to explore the therapeutic capacity of hMSCs and the precise mechanism involved in other related models, such as pancreatitis-associated lung injury. Another limitation is that we did not detect and analyze differences in expression level and effect among all the possible secreted therapeutic factors (from both human and animal sources) for a SAP animal model after treatment of hMSCs. Therefore, we referred to many previous related studies, but further studies will also be required to explore the relative contribution of each of these factors. Lastly, whether TSG-6 exerts its beneficial effects in SAP may also depend on the time, and was only observed at a relatively important time-point in our present study. Nevertheless, each study needs a certain time course and these limitations will be addressed in future studies.

In summary, our findings revealed that i.v.-administered hMSCs remarkably promote recovery from SAP, primarily by suppressing inflammation and decreasing pancreatic injury. The beneficial effects of hMSCs were shown to occur in the absence of significant engraftment of hMSCs in the damaged pancreas and were due primarily to their secretion of beneficial factors including the most critical anti-inflammatory cytokine TSG-6. Furthermore, hMSCs ameliorate SAP, at least in part, as a result of the suppressive effects of secreted TSG-6 on oxidative stress, NLRP3 inflammasome and NF-κB signaling dependent on CD-44 expression in pancreatic acinar cells that play a key role in initiating pancreatic inflammation and injury.

## Additional Information

**How to cite this article**: He, Z. *et al*. Intravenous hMSCs Ameliorate Acute Pancreatitis in Mice via Secretion of Tumor Necrosis Factor-α Stimulated Gene/Protein 6. *Sci. Rep.* **6**, 38438; doi: 10.1038/srep38438 (2016).

**Publisher's note:** Springer Nature remains neutral with regard to jurisdictional claims in published maps and institutional affiliations.

## Supplementary Material

Supplementary Figures
