# Supplementary material for: Intravenous hMSCs Ameliorate Acute Pancreatitis in Mice via Secretion of Tumor Necrosis Factor-α Stimulated Gene/Protein 6
Source: Sci Rep. 2016 Dec 5;6:38438. doi: 10.1038/srep38438 (PMC5137159; doi:10.1038/srep38438)
Supplement: Supplementary Figures [file srep38438-s1.pdf]

Intravenous hMSCs Ameliorate Acute Pancreatitis in Mice via Secretion of Tumor Necrosis Factor-α Stimulated Gene/Protein 6

Zhigang He, Jie Hua, Daohai Qian, Jian Gong, Shengping Lin, Chenglei Xu, Ge Wei, Hongbo Meng, Tingsong Yang, Bo Zhou, Zhenshun Song*

Zhigang He and Jie Hua contributed equally to this work.

Department of Hepatobiliary and Pancreatic Surgery, Shanghai Tenth People's Hospital, School of Medicine, Tongji University Shanghai, China.

Corresponding author: Zhenshun Song, M.D., Ph.D.

Postal address: Department of General Surgery, Shanghai Tenth People's Hospital, School of Medicine, Tongji University,

301 Yanchang Middle Road, Shanghai 200072, China.

Telephone: +86 137 6120 5962

Fax: +86 021 6630 7365

E-mail: zs_song@hotmail.com

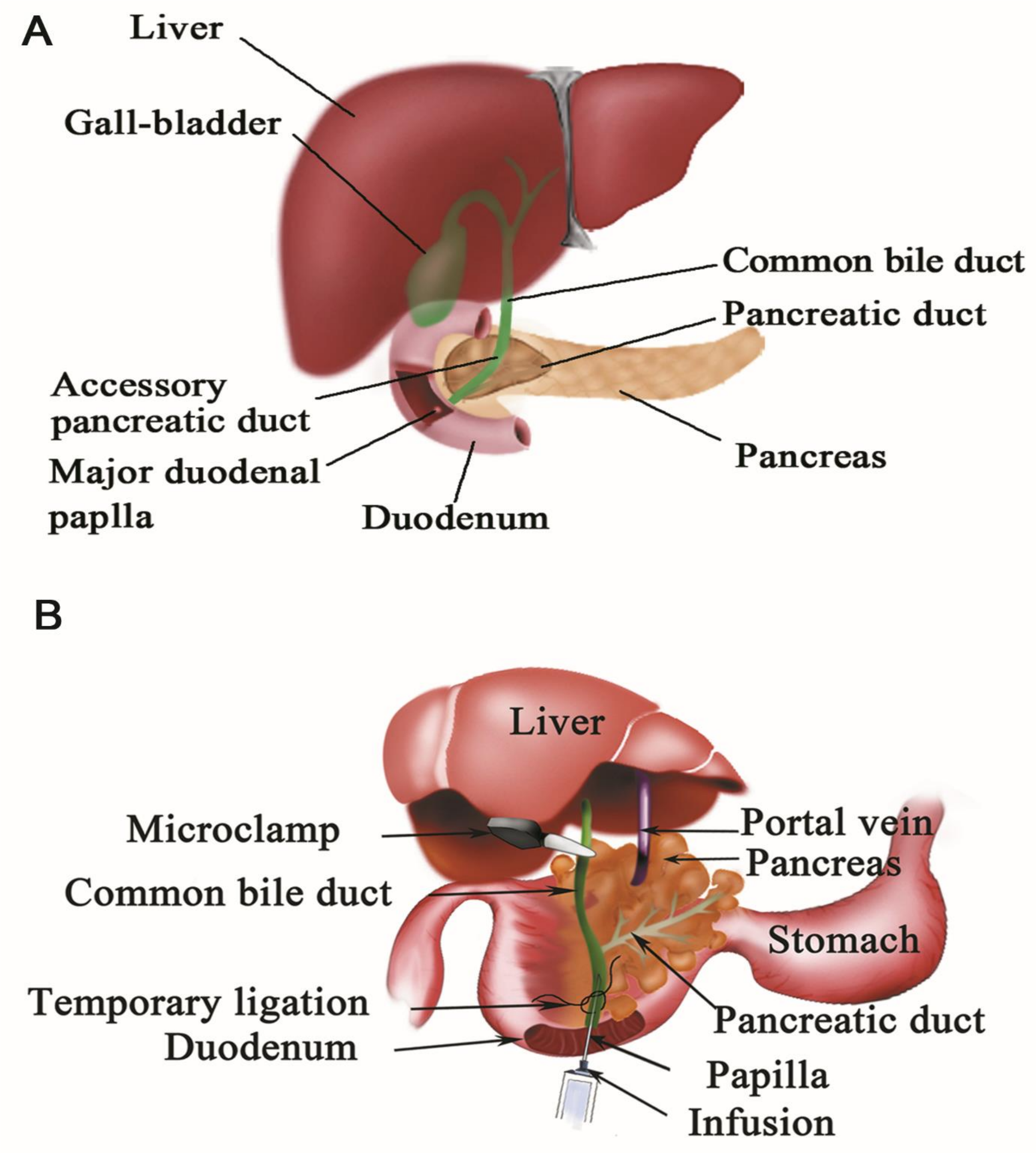

Figure S1. Establishment of a mouse model of experimental severe acute pancreatitis

(A) Gross normal anatomy of hepatobiliary and pancreas in the human. Humans and mice have some differences in the anatomical structure and distribution of the pancreas and pancreatic duct. (**B)** Gross anatomy of hepatobiliary ducts and pancreas in mice and the key process of establishing a SAP mouse model. With the aid of a

dissecting microscope, a temporary ligation was performed in the distal part of the common bile duct to prevent leakage of sodium taurocholate solution. Injection of the solution into the liver was prevented by placing a micro clamp on the proximal common bile duct. Sodium taurocholate solution was then retrogradely infused into the bile-pancreatic duct over a 10-minute period using an infusion pump.

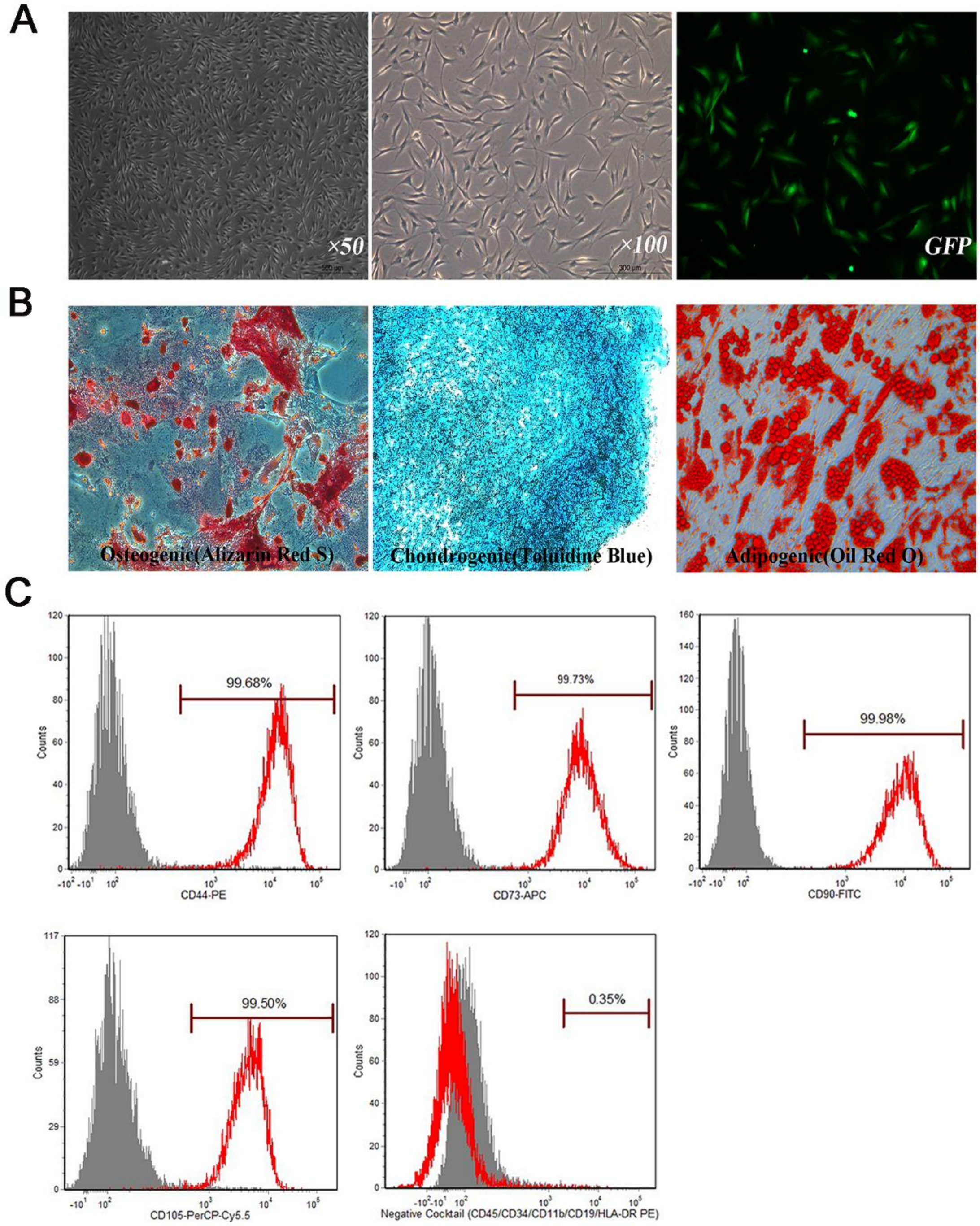

Figure S2. Characterization of hMSCs isolated from human bone marrow.

(A) Fibroblast-like morphology of hMSCs or hMSCs expressing GFP fluorescence on plastic culture dishes. **(B)** Multilineage differentiation capacity of hMSCs, osteogenic, chondrogenic and adipogenic

differentiation. **(C)** Surface marker expression of hMSCs analyzed by flow cytometry.
